# Analysis of *Plasmodium vivax* Apical Membrane Antigen-1 (PvAMA-1) Haplotypes among Iranian Isolates

**DOI:** 10.22088/BUMS.6.4.222

**Published:** 2017-11-16

**Authors:** Fatemeh Nedaei, Zahra Noormohammadi, Saied Reza Naddaf, Somayeh Mohammadi, Ahmad Reza Esmaeili Rastaghi

**Affiliations:** 1 *Department of Parasitology, Pasteur Institute of Iran, Tehran, Iran.*; 2 *Department of Biology* *, College of Basic Science* ***, *** *Islamic Azad University, Science and Research Branch, Tehran, Iran.*

**Keywords:** Apical membrane antigen-1 (AMA-1), DNA sequencing, genetic polymorphism, haplotype, malaria transmission, Plasmodium vivax

## Abstract

*Plasmodium vivax* apical membrane antigen-1(PvAMA-1) is a surface protein with polymorphic sites. This study was aimed to analyze the polymorphic amino acid residues at PvAMA-1 in different infected age groups. 92 blood samples were collected from the south and southeast of Iran. The DNA coding for the domain I (DI), DII, and partial DIII of this antigen was amplified by Nested-PCR, and sequenced. Nucleotide mutations were found in 49 sites and based on the amino acid sequence, 30 variable sites were detected. Age distribution of malaria cases showed that the majority of the patients were between 10 to 30 years old. The scattering plot haplotypes by age showed an increasing incidence rate with age during childhood, whereas, incidence was the lowest in patients under five years old. Comparison of the polymorphic sites of PvAMA-1 in Iranian isolates with those found in other geographic regions of the world indicated nine common variable positions. In addition, a significant dependence was found between some particular substitutions and age categories. Dependence between particular substitutions and age groups suggests that certain residues in AMA-1 are responsible for clinical attacks in different ages, likely as a result of host immune pressure. The crystal structure of the PvAMA-1 showed that the amino acid substitutions that changed the protein charge were exclusively located in loops and turns where, the interactions with antibodies could occur. These data provide the necessary information for an AMA-1 based malaria vaccine design to be effective across all ages.

Malaria is the most important infectious disease, and continues to be a major global health problem in the world (1). According to the latest report from World Health Organization (WHO) around 214 million cases of malaria were reported globally with an estimated 438,000 deaths (2). Of the five *Plasmodium* species causing malaria in humans, *P. vivax* is responsible for the most prevalent form of malaria outside Africa (3). Therefore, development of a vaccine against *P. vivax* is a necessary priority, particularly due to increased resistance of this parasite to the antimalarial drugs such as chloroquine, primaquine and pyrimethamine (4-7). Identification of target antigens that induce protective immune response is a pre-requisite for vaccine development. Many reports indicate apical membrane antigen1 (AMA-1) as one of the promising malaria vaccine candidates (8-11). AMA-1 is a type I integral membrane protein found in most of the Plasmodium species (11). AMA-1 expression is maximal in late schizogony during asexual reproduction in red blood cells (12, 13). It is initially located in merozoite apical organelles, and after processing relocates to the surface of mature merozoite (14). Around the time of erythrocyte invasion, the pro-sequence of AMA-1 is processed proteolytically by two c-terminal cleavages (15). The complete *P. vivax *AMA-1 (comprised of 562 amino acids) contains an ectodomain. Sixteen conserved cysteines contribute to 8 disulfide bonds, and divide the ectodomain into three subdomains (DI, DII, and DIII). The amino acid residues 43-248 make domain I, 249-385 make domain II, and 386-487 correspond to domain III (16). The stage specificity, and location of AMA-1 suggest that this protein is associated with the process of erythrocyte invasion as shown by the inhibition of parasite invasion using antibodies against AMA-1 (17). Also, generation of the parasites with disrupted *ama-1* gene by knockout technology suggests a critical role for AMA-1 in the development of the parasite during asexual blood-stage (18). Vaccination with AMA-1 can provide protective immunity in mice and monkeys, and induce antibodies that inhibit parasite development *in vitro*. These evidences introduce AMA-1 as a promising blood-stage vaccine candidate. In addition, unlike most other blood-stage proteins, *ama-1* gene, excluding some regions, is a conserved protein among various *Plasmodium* species. Point mutations are responsible for limited diversity in this antigen, and most of them are in DI (19, 20). Antigenic diversity is supposed to be a major mechanism for the parasite to escape from the host immune system, but remains the greatest obstacle in designing a malaria vaccine. Some findings show that protective immunity against AMA-1 and MSP-1 (merozoite surface protein) has a high degree of strain specificity (22, 23). Thus, AMA-1 polymorphism is a major problem to the efficacy of this protein as a vaccine component. On the other hand, some epitopes of this antigen produced by different allelic forms may be involved in the manifestation of clinical symptoms in particular age groups. Such variants should be considered to prepare repertories of AMA-1 allelic forms for developing a universal malaria vaccine. In the current study, the *P. vivax ama-1* gene was amplified and sequenced from 92 blood samples collected from two different malaria endemic regions of Iran. The study was conducted to analyze the population structure of *pvama-1* Iranian isolates as an important malaria vaccine candidate. Finally, this investigation introduces the common mutation sites that should be considered for vaccine design.

## Materials and methods

**Study area and sample collection**

A total of 92 blood samples were collected from two different malaria endemic regions of Iran (Hormozgan, and Sistan & Baluchestan provinces with the highest malaria incidence on the country (24)) during 2009-2011. The blood samples were obtained from patients with uncomplicated symptomatic malaria who have been referred to local health centers in these areas. The presence of *P. vivax* infection was diagnosed by the light microscopic analysis of Giemsa-stained thick, and thin blood smears. Two ml venous blood was collected from patients in tubes containing EDTA, and stored at -20 °C until used. Blood collections were made after obtaining informed consent from the patients or the parents/legal guardians of children. This investigation was approved by the Ethics Review Committee of Pasteur Institute of Iran.

**DNA extraction and PCR** **amplification** **of the P. vivax** **ama-1 gene**

Whole blood sample (200 μl) was used to isolate genomic parasite DNA using the QIAamp DNA Blood Mini Kit (Qiagen, Germany) according to the manufacturer’s protocol. Two-step PCR method was performed to amplify the *pvama-1* gene. The 1139 bp region of the *pvama-1* gene was initially amplified using set of primers PvAMAF1 (5′-GTTGAGAGAAGCACACGAATG-3′) and PvAMA2 (5′-GAGATAAATATCCTCGGCAGG-3′), and then the primary PCR product was used as template in a second round of PCR to amplify the internal 1000 bp region for which DI and DII are available, using the PvAMAF2 (5′-TGCAGAAGTGGAAAATGCAAAG-3′) and PvAMA4 (5′-CCATCAACACTGTACAGATTC-3′) primers. PCR cycles were performed as described previously (25), and the data are available in the GenBank database under the accession numbers KF181626-KF181642, and KF422636-KF422681.

**Sequence and statistical analyzes**

Nucleotide and amino acid sequences were aligned by MEGA program (26), with the Salvador-1 sequence (XM_001615397) as the reference strain. The haplotypes were classified based on the nucleotide sequences using DnaSP ver. 5. 10. 01 (27). Nucleotide mutations and amino acid variable sites were detected by the MEGA program. Nucleotide BLAST search was done to compare the *pvama-1* sequences in this study with the previously reported sequences in the Gen Bank data base. Only the sequences for which DI and DII were available were included in analysis. Comparative analysis of the variable sites at *pvama-1 *sequences was performed between 92 Iranian isolates, and the previously reported sequences from various geographic regions including India, Thailand, Venezuela and Sri lanka (28-32). The statistical analysis was performed using the two tailed Fisher's exact test in GraphPad program (http:// www.openepi.com/ oe2.3/ menu/ openepimenu.htm).

**Crystal structure of PvAMA-1**

Amino acid substitutions were shown on the 3D structure of PvAMA-1. The crystal structure was generated with Weblab Viewer Lite 4.2 (http:// www. scalacs.org/ TecherResources), and PDB ID 1w8k (16) was applied as the starting model.

## Results

***pvama-1*** **mutations and haplotypes**

Based on the sequencing data, a 911 bp region of the *ama-1* gene including the nucleotides 289 to 1199 was readable. This nucleotide sequence codes for amino acids 97 to 399 containing the major sequence of DI, complete DII, and partial DIII of AMA-1. The 92 amplicons sequenced were classified into 53 haplotypes (Table 1).

Nucleotide mutations were found in 49 sites in the first, the second and the third codon positions (15, 14, and 20, respectively). Variable amino acid sites 130N/K, 188K/N, and 242P/E are represented by third codon positions 390T/G, 564G/T, and 726 T/A that are also responsible for the generation of new parasite variants (Table 1).

**Haplotypes association with age of infection**

**Fig. 1:**
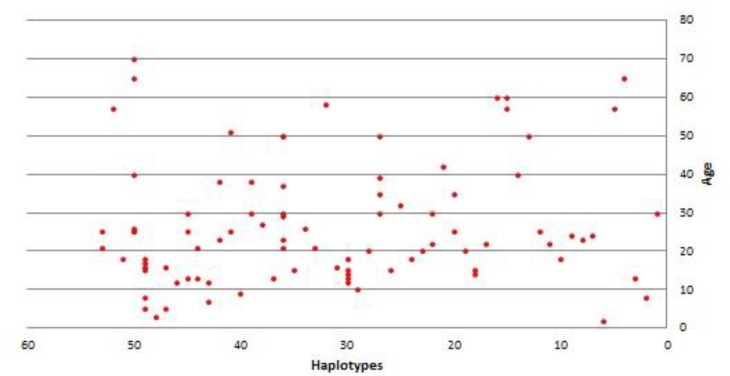
Distribution of haplotype frequency by age among 92 Iranian P .vivax isolates

The number of malaria cases was highest in the 20-30 as well as 10-20 age groups (Fig.1; Table2). Nonetheless, the percentage distribution of variants among age was not significantly different from that of the whole population. The most frequent haplotype was H18 (8.7%) distributed in 10-30 and 50-60 age categories, in a statistically non-significant manner (Table 2).

**Table 1:** Nucleotide polymorphisms in the pvama-1 gene among Iranian isolates (n=92).

f	306	318	320	321	335	336	351	352	359	390	394	411	418	422	434	564	565	567	568	577	628	680	682	683	684	726	758	829	831	863	877	968	1054	1068	1074	1075	1076	1078	1083	1092	1103	1106	1120	1125	1133	1138	1144	1145	1146	1148	1149	1151	1152	1154	1155	1158	1165	1172	1174	1176	1183	1187
Sal1 _	T	T	A	C	G	A	A	G	A	T	A	A	T	C	A	G	G	A	A	C	C	A	A	G	C	T	G	G	A	G	G	C	A	T	T	A	C	A	G	C	A	A	A	G	A	C	G	T	A	A	C	T	G	A	G	C	A	A	A	A	A	A
Hap-1 2	C	C	.	T	C	C	.	.	G	G	G	C	.	A	C	.	A	T	.	.	.	T	G	A	T	.	.	A	G	.	.	.	.	.	.	.	.	.	.	.	.	.	.	.	.	.	.	A	G	.	.	C	.	.	.	.	.	.	.	.	.	.
Hap-2 1	C	C	C	T	C	C	.	.	G	.	.	C	.	A	C	.	A	.	C	.	.	T	G	A	T	.	.	.	G	.	.	.	.	.	.	.	.	.	.	.	T	.	.	.	.	.	.	.	.	.	.	.	.	.	.	.	.	.	.	.	.	.
Hap-3 1	C	.	.	.	A	C	.	.	G	.	.	C	A	.	C	.	.	.	.	.	.	T	G	A	T	.	.	A	G	.	.	.	.	.	.	.	.	.	.	.	.	.	.	.	.	.	.	.	.	.	.	C	.	.	.	.	.	.	.	.	.	.
Hap-4 7	.	.	.	.	A	.	.	.	G	.	.	C	A	.	C	.	.	.	.	.	T	.	.	.	.	.	.	A	G	.	.	.	.	.	.	.	.	.	.	.	T	.	.	.	.	.	.	.	.	.	.	C	.	.	.	.	.	.	.	.	.	.
Hap-5 7	C	C	T	A	.	.	.	G	.	.	.	C	.	.	C	.	.	.	.	T	T	.	.	.	.	.	.	.	G	.	.	.	.	.	.	.	.	.	.	A	.	.	.	.	.	A	.	.	.	.	.	C	.	.	.	.	.	.	.	.	.	.
Hap-6 1	C	.	.	.	C	C	.	.	G	.	G	C	A	.	C	.	A	T	.	.	.	.	.	.	.	.	.	A	G	.	.	.	.	.	.	.	.	.	.	.	.	.	.	.	.	.	.	.	.	.	.	C	.	.	.	.	.	.	.	.	.	.
Hap-7 2	C	C	T	C	C	.	.	G	G	G	C	.	.	C	.	.	.	.	T	T	.	.	.	.	.	.	A	G	.	.	.	.	.	.	.	.	.	.	.	.	T	.	.	.	.	.	.	.	.	.	.	C	.	.	.	.	.	.	.	.	.	.
Hap-8 1	C	C	C	T	C	C	.	.	G	.	.	C	.	A	C	.	A	T	.	.	.	.	.	.	.	.	.	A	G	A	.	.	.	.	.	.	.	.	.	.	T	.	.	.	.	.	.	.	.	.	.	C	.	.	.	.	.	.	.	.	.	.
Hap-9 3	.	.	.	.	A	.	.	.	G	.	.	C	A	.	C	.	.	.	.	.	.	.	.	.	.	.	.	.	G	.	.	.	.	.	.	.	.	.	.	.	.	.	.	.	.	.	.	.	.	.	.	.	.	.	.	.	.	.	.	.	.	.
Hap-10 2	.	.	.	.	A	.	.	.	G	.	.	C	A	.	C	.	.	.	.	.	T	.	.	.	.	.	.	A	G	.	.	.	.	.	.	.	.	.	.	.	.	.	.	.	.	.	.	.	.	.	.	C	.	.	.	.	.	.	.	.	.	.
Hap-11 2	.	.	.	.	A	.	.	.	G	.	.	C	.	A	C	.	A	.	.	.	.	T	G	A	T	.	.	A	G	A	.	.	G	.	.	.	.	.	.	.	.	.	.	.	.	.	.	.	.	.	.	.	.	.	.	.	.	.	.	.	.	.
Hap-12 2	C	C	C	T	C	C	.	.	G	.	G	C	.	A	C	T	A	.	.	.	.	.	.	.	.	.	.	.	G	.	.	.	.	.	.	.	.	.	.	.	.	.	.	.	.	.	.	.	.	.	.	.	.	.	.	.	.	.	.	.	.	.
Hap-13 2	C	C	C	T	.	.	.	.	G	.	G	C	.	.	.	.	.	.	.	.	.	.	.	.	.	.	.	A	G	.	.	.	.	.	.	.	.	.	.	.	T	.	.	.	.	.	.	.	.	.	.	C	.	.	.	.	.	.	.	.	.	.
Hap-14 1	C	C	.	T	A	.	.	.	G	.	.	C	.	.	C	.	.	.	.	T	T	.	.	.	.	.	.	.	G	.	.	.	.	.	.	.	.	.	.	A	.	.	.	.	.	A	.	.	.	.	.	C	.	.	.	.	.	.	.	.	.	.
Hap-15 2	.	.	.	.	.	.	.	.	.	.	.	C	.	.	C	.	A	.	G	.	T	T	G	A	T	.	.	A	G	.	.	.	.	.	.	.	.	.	.	.	.	.	.	.	.	.	.	.	.	.	.	.	.	.	.	.	.	.	.	.	.	.
Hap-16 1	C	C	.	T	C	C	.	.	G	G	G	C	.	A	C	.	.	.	.	T	T	.	.	.	.	.	.	A	G	.	.	.	.	.	.	.	.	.	.	.	.	.	.	.	.	.	.	.	.	.	.	C	.	G	A	T	.	.	.	.	.	G
Hap-17 1	.	.	.	.	A	.	.	.	G	.	.	C	A	.	C	.	.	.	.	.	T	T	T	A	T	.	.	A	G	A	.	.	G	.	.	.	.	.	.	.	.	.	.	.	.	.	.	.	.	.	.	.	.	.	.	.	.	.	.	.	T	.
Hap-18 8	.	.	.	.	A	.	.	.	G	.	.	C	A	.	C	.	.	.	.	.	T	T	G	A	T	.	.	A	G	A	.	.	G	.	.	.	.	.	.	.	.	.	.	.	.	.	.	.	.	.	.	.	.	.	.	.	.	.	.	.	.	.
HaP-19 1	C	C	.	T	C	C	.	.	G	G	G	C	.	A	C	.	A	T	.	.	.	T	G	A	T	.	.	A	G	.	.	.	.	.	.	.	.	.	.	.	.	.	.	.	.	.	.	A	.	.	.	C	.	.	.	.	.	.	.	.	.	.
Hap-20 1	.	.	.	.	A	.	.	.	G	.	G	C	A	.	C	.	A	T	.	.	.	.	.	.	.	.	.	A	G	.	.	.	.	.	.	.	.	.	.	.	.	.	.	.	.	.	.	A	G	.	.	C	.	.	.	.	.	.	.	.	.	.
Hap-21 1	.	.	.	.	A	.	.	.	G	.	.	C	.	.	C	.	.	.	.	.	T	T	.	A	T	.	.	A	G	.	.	.	.	.	.	.	.	.	.	.	.	.	.	.	.	.	.	.	.	.	.	C	.	.	.	.	.	.	.	.	.	.
Hap-22 1	C	C	T	T	A	.	.	.	.	.	.	C	.	.	C	.	A	.	G	.	.	T	G	A	T	.	.	A	G	.	.	.	.	.	.	.	.	.	.	.	.	.	.	.	.	.	.	.	.	.	.	C	.	.	.	.	.	.	.	.	.	.
Hap-23 1	C	C	.	T	C	C	.	.	G	G	G	C	.	A	C	.	A	.	.	.	.	T	.	A	T	.	.	A	G	.	.	.	.	.	.	.	.	.	.	.	.	.	.	.	.	A	A	.	.	.	.	C	.	.	.	.	.	.	.	.	.	.
Hap-24 5	C	C	C	T	C	C	.	.	G	.	G	C	.	A	C	.	A	T	.	.	.	T	G	A	T	.	.	A	G	.	.	.	.	.	.	.	.	.	.	.	.	.	.	.	.	.	.	.	.	.	.	C	.	.	.	.	.	.	.	.	.	.
Hap-25 1	.	.	.	.	.	.	.	.	.	.	.	C	.	.	.	.	A	.	C	.	.	T	G	A	T	.	.	.	G	.	.	G	.	.	.	.	.	.	.	.	.	.	.	.	.	A	.	.	.	.	.	C	.	.	.	.	.	.	.	.	.	.
Hap-26 1	C	C	C	T	C	C	.	.	G	.	.	C	A	.	C	.	.	.	.	.	.	.	.	.	.	.	.	.	G	.	.	.	.	.	.	.	.	.	.	.	.	.	.	.	.	.	.	.	.	.	.	.	.	.	.	.	.	.	.	.	.	.
Hap-27 4	C	C	.	T	C	C	.	.	G	G	G	C	.	A	C	.	A	.	C	.	.	T	G	A	T	.	.	A	G	.	.	.	.	.	.	.	.	.	.	.	T	.	.	.	.	.	.	.	.	.	.	C	.	.	.	.	.	.	.	.	.	.
Hap-28 1	C	C	C	T	C	C	.	.	G	.	G	C	.	A	C	.	A	.	.	.	.	T	.	A	T	.	.	A	G	.	.	.	.	.	.	.	.	.	.	.	.	.	.	.	.	.	.	.	.	.	.	C	.	.	.	.	.	.	.	.	.	.
Hap-29 1	C	C	.	T	C	C	.	.	G	G	G	C	.	A	C	.	A	T	.	.	.	T	.	A	T	.	.	A	G	.	.	.	.	.	.	.	.	.	.	.	.	.	.	.	.	.	.	A	G	.	.	C	.	.	.	.	.	.	.	.	.	.
Hap-30 1	C	C	C	T	A	.	.	.	G	.	.	C	A	.	C	.	.	.	.	.	T	.	.	.	.	.	.	A	G	.	.	.	.	.	.	.	.	.	.	.	T	.	.	.	.	.	.	.	.	.	.	C	.	.	.	.	.	.	.	.	.	.
Hap-31 1	C	C	C	T	A	.	.	.	G	.	.	C	.	.	C	.	.	.	.	.	.	.	.	.	.	.	.	A	G	.	.	.	.	.	.	.	.	.	.	.	.	.	.	.	.	.	.	.	.	.	.	C	.	.	.	.	.	.	.	.	.	.
Hap-32 1	.	.	.	.	A	.	.	.	G	.	.	C	A	.	C	.	A	.	G	.	T	T	G	A	T	.	.	A	G	.	.	.	.	.	.	.	.	.	.	.	T	.	.	.	.	.	.	.	.	.	.	G	.	.	T	.	.	.	.	.	.	.
Hap-33 1	C	.	.	.	A	.	.	.	G	.	.	C	A	.	C	.	.	.	.	.	.	T	A	T	.	.	A	G	A	.	.	.	.	.	.	.	.	.	.	.	.	.	.	.	.	.	.	.	.	.	.	C	.	.	.	.	.	.	.	.	.	.
Hap-34 1	.	.	.	.	A	.	.	.	G	.	.	C	A	.	C	.	.	.	.	.	T	.	.	.	.	.	A	.	G	.	.	.	.	.	.	.	.	.	.	.	.	.	.	.	.	.	.	.	.	.	.	C	.	.	.	.	.	.	.	.	.	.
Hap-35 1	C	C	C	T	A	.	.	.	G	.	.	C	A	.	C	.	.	.	.	.	.	.	.	.	.	.	.	.	G	.	.	.	.	.	.	.	.	.	.	.	.	.	.	.	.	.	.	.	.	.	.	.	.	.	.	.	.	.	.	.	.	.
Hap-36 2	.	.	.	.	A	.	.	.	G	.	.	C	A	.	C	.	.	.	.	.	.	.	.	.	.	.	.	A	G	.	.	.	.	C	.	.	.	.	.	.	.	.	.	.	.	.	.	A	G	.	.	C	.	.	.	.	.	.	.	.	.	.
Hap-37 1	.	.	.	.	A	.	.	.	G	.	.	C	.	A	C	.	A	.	.	.	.	T	G	A	T	A	.	A	G	A	.	.	G	.	.	.	.	.	.	.	.	.	.	.	.	.	.	.	.	.	.	.	.	.	.	.	.	.	.	.	.	.
Hap-38 1	.	.	.	.	A	.	.	.	G	.	.	C	A	.	C	.	.	.	.	.	T	.	.	.	.	.	.	A	G	.	.	.	.	.	A	T	A	C	.	.	T	T	C	.	.	.	.	.	.	.	.	C	.	.	.	.	.	.	.	.	.	.
Hap-39 2	C	C	C	T	C	C	.	.	G	.	G	C	.	A	.	.	A	.	.	.	.	.	.	A	T	.	.	A	G	.	.	.	.	.	.	.	.	.	.	.	T	.	.	.	.	.	.	A	.	.	.	C	.	.	.	.	.	.	.	.	T	T
Hap-40 1	C	C	.	T	C	C	.	.	G	G	G	C	.	A	C	.	A	.	C	.	.	T	G	A	T	.	.	A	G	.	.	.	.	.	.	.	.	.	.	.	.	.	.	.	G	.	.	.	.	.	.	C	.	.	.	.	.	.	.	.	.	.
Hap-41 1	C	C	.	T	C	C	.	.	G	G	G	C	.	A	C	.	A	.	C	.	.	T	G	A	T	.	.	A	G	.	.	.	.	.	.	.	.	.	.	.	.	.	C	.	.	.	.	.	.	.	.	.	.	.	.	.	.	.	.	.	T	T
Hap-42 1	.	.	.	.	A	.	.	.	G	.	G	C	A	.	C	.	A	T	.	.	.	.	.	.	.	.	.	A	G	.	.	.	.	.	.	.	.	.	.	.	.	.	.	.	.	.	.	A	G	.	.	C	.	.	.	.	C	.	.	.	T	T
Hap-43 1	.	.	.	.	A	.	.	.	G	.	G	C	A	.	C	.	A	T	.	.	.	.	.	.	.	.	.	A	G	.	.	.	.	.	.	.	.	.	.	.	.	.	.	.	C	.	.	A	.	G	A	C	C	G	A	T	C	.	.	.	T	.
Hap-44 1	C	C	.	.	A	.	.	.	G	.	.	C	.	.	C	.	.	.	.	.	T	.	.	.	.	.	.	A	G	.	.	.	.	.	.	.	.	.	.	.	.	.	.	.	.	.	.	.	.	.	.	C	.	.	A	.	C	T	.	G	.	.
Hap-45 1	C	C	.	.	C	.	.	.	G	.	.	C	.	.	C	.	.	.	.	.	T	.	.	A	.	.	.	A	G	.	.	.	.	.	.	.	.	.	.	.	.	.	.	C	.	.	.	.	.	.	.	C	.	.	.	.	.	.	.	.	.	.
Hap-46 1	C	C	C	T	A	.	T	A	G	.	G	C	.	.	.	.	.	.	.	T	T	.	.	.	.	.	.	A	G	.	.	.	.	.	.	.	.	.	.	.	T	.	.	.	.	.	.	.	.	.	.	C	.	.	A	.	.	.	T	G	.	.
Hap-47 1	.	.	.	.	A	.	.	.	G	.	G	C	A	.	C	.	A	T	.	.	.	.	.	.	.	.	.	A	G	.	.	.	.	.	.	.	.	.	.	.	.	.	.	.	.	.	.	A	G	.	.	C	.	.	.	.	.	T	.	.	T	.
Hap-48 1	C	C	C	T	A	.	.	.	G	.	.	C	.	.	C	.	.	.	.	T	T	.	.	.	.	.	.	.	G	.	.	.	.	.	.	.	.	.	.	A	.	.	.	.	.	A	.	.	.	.	.	C	.	.	.	.	.	.	.	.	T	.
Hap-49 1	C	C	C	T	.	.	.	.	G	.	G	C	.	.	.	.	A	.	G	.	T	T	G	A	T	.	.	A	G	.	.	.	.	.	.	.	.	.	.	.	.	.	.	.	.	.	.	.	.	.	.	C	.	.	.	.	.	.	.	G	T	.
Hap-50 1	.	.	.	.	.	.	.	.	.	.	.	C	.	.	.	.	A	.	C	.	.	T	G	A	T	.	.	.	G	.	A	.	.	.	.	.	.	.	A	.	.	.	.	T	.	.	A	.	.	.	.	C	.	.	.	.	.	.	.	.	.	.
Hap-51 1	C	C	C	T	C	C	.	.	G	.	G	C	.	A	C	.	A	T	.	.	.	T	G	A	T	.	.	A	G	.	.	.	.	.	.	.	.	.	.	.	.	.	.	.	.	.	.	.	.	.	.	C	.	.	.	.	.	.	.	.	T	.
Hap-52 1	C	C	.	T	A	.	.	.	G	.	.	C	.	A	C	.	.	.	.	.	.	.	.	A	.	.	.	A	G	.	.	.	.	.	.	.	.	.	.	.	.	.	.	.	.	.	.	.	.	.	.	C	.	.	.	.	.	.	.	.	T	.
Hap-53 1	.	.	.	.	A	.	.	.	G	.	.	C	A	.	C	.	.	.	.	.	.	.	.	.	.	.	.	A	G	.	.	.	.	.	.	.	.	.	.	.	.	.	.	.	.	.	.	.	.	.	.	.	.	.	A	.	.	.	.	.	T	.
.	.	.	.	.	.	.	.	.	.	.	.	.	.	A	G	.	.	C	.	.	.	.	.	.	.	.	.	.																																		.

**Table 2:** Distribution of malaria cases and haplotype frequency by different age categories among 92 Iranian PvAMA-1 isolates

1-10	9.78	H5 (×3), H6, H7, H11, H14, H48, H52
10-20	27.17	H3, H5 (×4), H7, H8, H9, H10, H11, H17, H19, H23, H24 (×5), H25, H28, H30, H36 (×2), H44, H51
20-30	28.26	H1(×2), H4 (×3), H9, H10, H12, H13, H16, H18 (×3), H20, H21, H26, H31, H32, H34, H35, H37, H42, H43, H45, H46, H47
30-40	15.21	H9, H12, H15 (×2), H18 (×3), H27 (×3), H29, H32, H34, H53
40-50	4.34	H4 (×2), H33, H40
50-60	9.78	H2, H13, H18 (×2), H22, H27, H39, H41, H49
60-70	5.43	H4 (×2), H38, H39, H50
Total (%)	100	

**PvAMA-1 variants**

**Fig. 2:**
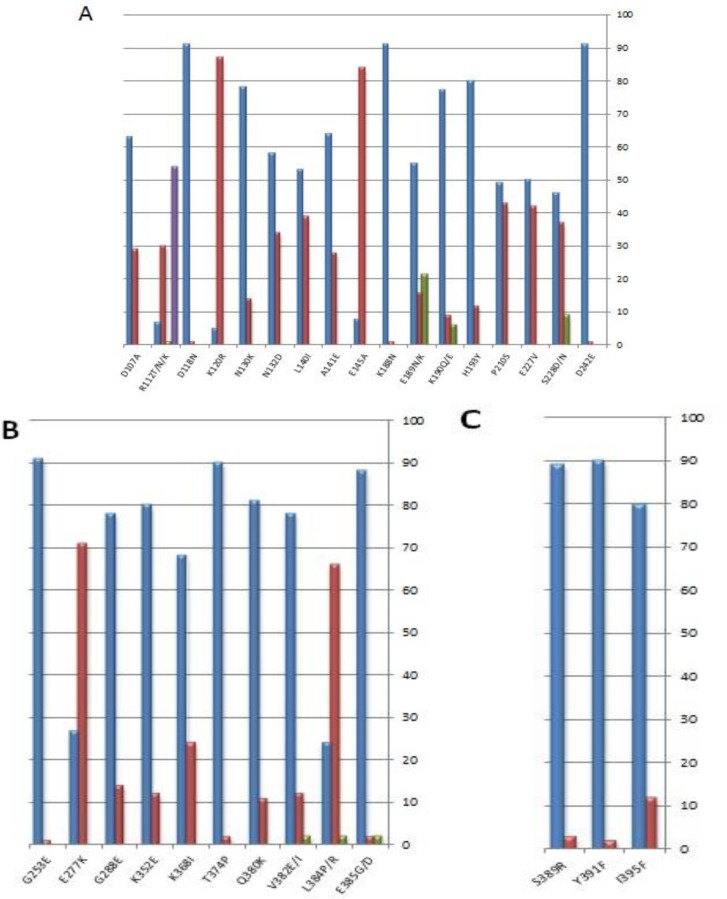
The frequency of amino acid residues of each polymorphic position at PvAMA-1 isolates from Iran (n=92). A: Domain I; B: Domain II; C: Domain III

Based on the amino acid sequences, 30 variable sites were detected, 23 of them were dimorphic, 6 were trimorphic and the position 112 showed four alternative amino acid residues (R, T, N, K, tetramorphic). The positions 112, 189, 190, 228, 382, 384 and 385 at DI and DII of the PvAMA-1 included substitutions with more than two possible amino acid residues (Fig. 2). Some of the amino acid substitutions that occurred in the segment caused changes in the charge of the protein. These consisted of Q189K, K190Q substitutions in DI, and Q277K, K352Q substitutions in DII of PvAMA-1 where they could affect the protein structure. Although, some amino acid substitutions with no effect on the protein charge were K120R, and D242E in AMA-1 DI. Identified patterns of the mutation are one of the most important strategies used by the malaria parasites to escape from the host immune system and should be considered for vaccine design.

Comparative analysis of the polymorphic sites in PvAMA-1 between Iranian isolates and those previously reported from the other malaria endemic areas (India, Thailand, Venezuela and Sri lanka) (28-32) revealed that 9 of these variable sites were common (Table 3) including 107 D/A, 112R/T/N/K, 189E/N/K, 190K/Q/E, 210P/S in DI, 288G/E, 352K/E, 380Q/K in DII, and 384L/P/R in DIII of the PvAMA-1. 

**Association of PvAMA-1 variants with age of infection**

In order to evaluate the effect of polymorphic amino acid positions in the AMA-1 on age of infection, the rate of amino acid residues at mutation sites shared between Iran, India, Thailand, Venezuela and Sri Lanka was assessed. The results showed a strong association between the rate of amino acid residues Q and P in position 384, and clinical attacks in 30-40 age group. Similarly, P384, D107, K189, K190, and L384 positions were associated with malaria incidence in over 40 years (P<0.05, Table 4).

**Table 3:** Comparative analyzes of variable sites at PvAMA-1 between Iranian isolates and the previously reported sequences in Genbank database

Sri Lanka (n=23)^d^		Venezuela (n=40)^c^		Thailand (n=60)^b^		India (n=72)^a^		Iran (n=92)		
f	mutations	f	mutations	f	mutations	f	mutations	f	mutations	Position
_	Q	_	Q	43/17	Q/H	_	_	_	_	25
_	G	33/7	G/V	_	G	_	_	_	_	42
_	T	30/10	T/S	_	T	_	_	_	_	44
_	R	14/26	R/K	_	R	_	_	_	_	66
**19/3/1**	**D/A/N**	**8/32**	**D/A**	**1/59**	**D/A**	**41/30/1**	**D/A/N**	**63/29**	**D/A**	**107**
**1/22**	**T/K**	**6/28/5/1**	**R/T/K/S**	**42/18**	**T/K**	**10/27/1/34**	**R/T/N/K**	**7/30/1/54**	**R/T/N/K**	**112**
_	D	_	D	_	D	71/1	D/N	91/1	D/N	118
_	R	33/7	R/K	42/18	K/R	2/69/1	K/R/S	87/5	R/K	120
_	N	26/14	N/K	_	N	64/8	N/K	78/14	N/K	130
22/1	N/D	26/14	N/D	_	N	36/36	N/D	58/34	N/D	132
18/5	I/L	29/11	L/I	_	I	40/32	L/I	53/39	L/I	140
20/3	A/E	18/22	A/E	_	A	58/14	A/E	64/28	A/E	141
_	A	25/15	A/E	_	A	54/18	A/E	84/8	A/E	145
22/1	K/N	_	K	_	K	70/2	K/N	91/1	K/N	188
**17/6**	**E/K**	**24/16**	**E/K**	**17/43**	**E/K**	**31/7/34**	**E/N/K**	**55/16/21**	**E/N/K**	**189**
**20/3**	**K/E**	**24/16**	**K/Q**	**17/43**	**K/E**	**53/5/14**	**K/Q/E**	**77/9/6**	**K/Q/E**	**190**
21/2	H/Y	35/5	H/Y	_	H	6/66	H/Y	80/12	H/Y	193
**9/14**	**P/S**	**34/6**	**P/S**	**42/18**	**P/S**	**35/37**	**P/S**	**49/43**	**P/S**	**210**
22/1	V/L	_	V	43/17	V/L	_	V	_	V	218
16/7	E/V	_	E	_	E	44/28	E/V	50/42	E/V	227
16/7	S/D	_	S	_	S	44/28	S/D	46/37/9	S/D/N	228
_	D	_	D	_	D	_	D	91/1	D/E	242
19/4	G/E	_	G	_	G	71/1	G/E	91/1	G/E	253
14/9	K/E	39/1	K/E	_	K	61/11	K/E	71/27	K/E	277
**18/5**	**G/E**	**35/5**	**G/E**	**18/42**	**G/E**	**63/9**	**G/E**	**78/14**	**G/E**	**288**
**17/2/4**	**K/E/N**	**32/8**	**K/N**	**43/17**	**K/N**	**63/6/3**	**K/E/N**	**80/12**	**K/E**	**352**
12/11	K/I	33/7	K/I	_	K	60/12	K/I	68/24	K/I	368
22/1	F/L	_	F	_	F	_	F	_	F	370
_	T	_	T/S	_	T	_	T	90/2	T/P	374
**17/3/3**	**Q/K/R**	**33/7**	**Q/K**	**59/1**	**Q/R**	**69/2/1**	**Q/K/R**	**81/11**	**Q/K**	**380**
_	V	_	V	_	V	64/8	V/E	78/12/2	V/E/I	382
**9/7/7**	**L/P/R**	**18/22**	**L/P**	**1/59**	**L/R**	**38/19/15**	**L/P/R**	**24/66/2**	**L/P/R**	**384**
16/7	E/D	_	E	_	E	61/1/10	E/Q/D	88/2/2	E/G/D	385
_	S	_	S	_	S	_	_	89/3	S/R	389
_	Y	_	Y	_	Y	_	_	90/2	Y/F	391
_	I	_	I	_	I	_	_	80/12	I/F	395
20/3	K/R	_	K	_	K	_	_	_	_	400
20/3	H/R	12/28	H/R	_	H	_	_	_	_	438
_	N	_	N	43/17	N/D	_	_	_	_	445

a Thakur et al. (2008) (28); Rajesh et al. (2007) (29).

b Putaporntip et al. (2009) (30).

C Ord et al. (2008) (31).

d Gunasekera et al. (2007) (32).

**Fig. 3:**
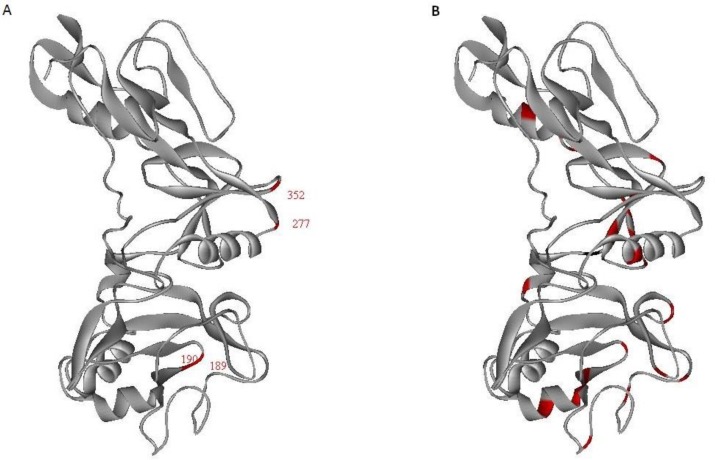
The surface representation of the mutation sites. Data was generated with ribbon model in Weblab Viewer Lite 4.2 program. Red color shows the amino acid substitutions and all other positions are gray color. A: mutations that changed the charge of the antigen (E189K, K190E, E277K and K352E); B: other polymorphic sites

Mapping of the amino acid substitutions on 3D structure of PvAMA-1 showed that mutations with changes in the charge of the protein were exclusively located in loop and turn secondary structures whereas, other mutation sites had been spread in the molecule (Fig. 3).

## Discussion

The present study was aimed to investigate the associations between the incidence of PvAMA-1 variants and age of infection.

The data show that the incidence of malaria reached the peak in the late teenage years, and declined with age in adulthood (Table 2; Fig. 1). This age distribution confirms that in populations with low malaria transmission, the incidence increases with age in teenagers and then declines; whereas in areas exposed to a very low level of transmission or to epidemic malaria, the clinical attack occurs across all ages (33). The decrease in malaria intensity with age in adults in the present study is in agreement with the study on *P. falciparum* in Kwazulu Natalin South Africa (34), and another report on *P. vivax* infection by Denis-lozano et al. (35) in Mexico that can be explained by the low level of transmission intensity in these areas. Besides, adults may have had immunity early in life due to previous exposure to the malaria parasite. Our findings are in contrast with the report of Baird et al. (36) in Irian Jaya which showed an increase in malaria infection with age in a population of adults from Java after migration to a hyper endemic area. However, the examined population in the present study has been exposed to a lower level of transmission than the population in the Irian Java study. The scattering plot shows an increasing incidence rate with age during childhood, whereas incidence is the lowest in patients under five years old. One possible explanation is that the children under 5 years old are commonly indoor at night, and are protected against mosquito bites. As children grow older, they may be more at risk of infective mosquito bites. Though, it is improbable that adults are at lower risk of infective bites than teenagers. The decline in malaria incidence in adults might explain that the infective bites in early childhood can be responsible for clinical protection in adults (33, 37).

**Table 4:** Age- specific patterns of amino acid substitutions at PvAMA-1 in Iranian isolates.

**384**			**380**			**352**			**288**		**210**		**190**			**189**			**112**					**107**				
R	P	L	R	K	Q	N	E	K	E	G	S	P	E	Q	K	K	N	E	S	K	N	T	R	N	A	D	f	Age
2.1	71.7	26	_	11.9	88	_	13	86.9	15.2	84.7	46.7	53.2	6.5	9.7	83.6	22.8	17.3	59.7	_	58.6	1	32.6	7.6	_	31.5	68.4	92	Iran
_	**85.7** *	**14.2** *	_	**42.8** *	**57.1** *	_	14.2	85.7	14.2	85.7	57.1	42.8	_	_	**100** *	14.2	14.2	71.4	_	71.4	_	28.5	_	_	28.5	71.4	9	0-10
_	**87.5** *	**12.5** *	_	18.7	81.2	_	6.2	93.7	12.5	87.5	37.5	62.5	_	6.2	**93.7** *	18.7	25	56.2	_	43.7	6.2	43.7	6.2	_	37.5	62.5	25	10-20
4.7	66.6	28.5	_	_	**100** *	_	9.5	90.4	9.5	90.4	42.8	57.1	4.7	_	**95.2** *	14.2	23.8	61.9	_	71.4	_	23.8	4.7	_	28.5	71.4	26	20-30
11.1	**33.3** **	**55.5** *	_	_	**1.0** **	_	11.1	88.8	11.1	88.8	44.4	55.5	**22.2** *	11.1	**66.6** *	**44.4** *	11.1	**44.4** *	_	55.5	_	33.3	11.1	_	**11.1** *	**88.8** *	14	30-40
_	**100** **	_	_	_	**100** *	_	_	**100** *	**33.3** *	**66.6** *	33.3	66.6	_	**33.3** *	**66.6** *	33.3	_	66.6	_	66.6	_	33.3	_	_	_	100*	4	40-50
_	62.5	37.5	_	_	**100** *	_	12.5	87.5	12.5	87.5	**25** *	**75** *	**25** *	**37.5** *	**37.** 5**	**75** **	_	**25** *	_	**25** *	_	**50** *	**25** *	_	**62.5** *	**37.5** **	9	50-60
_	**100** **	_	_	_	**100** *	_	_	**100** *	_	**100** *	50	50	_	25*	75	**50** *	_	50	_	50	_	25	**25** *	_	25	75	5	60-70

The polymorphic amino acid residues of each mutation site in the studied age groups were compared with that of the whole population by the two tailed Fisher exact test in Graph pad program.

*: significant values with P<0.05;

**: significant values with P< 0.0001

Therefore, in areas with high malaria transmission, some adults are less at risk for the disease due to the immunity acquisition occurrence prior in the life. In addition, it is possible that misdiagnosis has led to the decline in incidence recorded in young children. Misdiagnosed children may possess a measure of clinical tolerance by antimalarial therapy which has unlikely increased the malaria incidence with age in children. The existence of real differences in the reported malaria cases in different age categories would therefore support the concept of the clinical immunity hypothesis in some age categories (34).

Some amino acid substitutions found in AMA-1 are thought to be associated with disrupting epitopic targets of the immune system. Changes in amino acid charge from positive to negative or vice versa have a drastic effect on epitope detection by antibodies (21). In the present study, such replacements have occurred in four amino acid positions including Q189K, K190Q substitutions at Domain I, and Q277K, K352Q G substitutions at Domain II (Fig. 2 A and B) which were also found in India, Venezuela and Sri Lanka (Table 3).

Despite this, the Q277K replacement has not been found in Thai population that may be explained by the lower host immune selection pressure due to very low malaria incidence rate (30) and/or insufficient time to generate new alleles bearing this mutation. Moreover, some mutations involved in the amino acid changes with the same charge may be associated with functional constraints of the protein (K120R, D242E in AMA-1 Domain I) (Fig. 2 A).

Comparison of the polymorphic positions between five different populations showed that 9 positions were common (Table 3). The conservation of the common mutations suggests that these particular substitutions are necessary for the parasite to escape from the host immune system (21). If other mutations could play a similar role in the destruction of epitopes targeted by the immune system, different mutations would probably be expected between separate populations as shown in various haplotype repertoires.

The distribution of polymorphic amino acid residues (common between 5 comparative regions) by age categories (Table 4) indicated a strong age dependence between the frequency of Q and P residues in position 384, and clinical disease in 30-40 age group. Additionaly, a similar situation was found in P384, D107, K189, K190, and L384 positions in age 40 and over. In another study performed on *P. falciparum *AMA-1, Cortés et al. showed that residues E187 and E243 were strongly associated with malaria incidence in children under 10 years of age (21).

This strain-dependent incidence suggests that certain residues at particular positions decrease the AMA-1 immunogenicity, and a long exposure is necessary for inducing an effective immune response. These positions were assumed to be effective in controlling growth of the malaria parasite (21).

Furthermore, these findings show that particular amino acid substitutions could be responsible for the clinical attacks in different age groups, and there is an age-related selection in the immune system against certain amino acids at particular positions of the parasite’s AMA-1. In addition, the clinical presentation of the malaria infections is associated with different AMA-1 strains, suggesting that parasite factors are involved. Correspondingly, it was proposed that the higher morbidity in *P. falciparum* species could be related to the presence of certain residues in AMA-1(21).

Concerning the effect of certain amino acid residues on malaria infection rates, we cannot determine if these residues affect directly or their cooperation with other AMA-1 residues involved. yet, as the amino acid residues associated with the clinical malaria cases occur in multiple AMA-1 sequences, few parasite transmission can be ruled out. Thus, it is improbable that the clinical attacks are due to a combination of particular residues in other loci.

Knowledge of the amino acid substitutions, particularly those with charge *conversion*, could help to recognize the main epitopes that elicit protective antibodies*. *In the present study, these substitutions were *exclusively* observed in turn and loop secondary structures of the antigen (Fig. 3). Turns and loops generally lie on the surface of the proteins where they can participate in interactions between proteins and other molecules such as antibodies (38, 39).

The present study provides the necessary information to design a malaria vaccine to be effective in different age categories, and suggests that some of the epitopes in AMA-1 providing protective immunity response are strain specific. This problem is one of the difficulties in designing efficient vaccine based on only one allelic form of the protein, and was confirmed by previous reports (17, 19, 22, 40). One possible strategy to develop a universal malaria vaccine would be the inclusion of a large number of variants of the protein. Nonethe less, this strategy might be difficult for vaccine design. Therefore, knowledge of the sequences observed in the population to detect which of the variants may induce the protective immunity, could help to design an AMA-1-based vaccine. This study introduces 9 variable sites common between Iran, Thailand, Venezuela and Sri lanka including 107 D/A, 112R/T/N/K, 189E/N/K, 190K/Q/E, 210P/S in DI, 288G/E, 352K/E, 380Q/K in DII, and 384L/P/R in DIII of the PvAMA-1, that could be used as components of a polyvalent malaria vaccine based on PvAMA-1.

## References

[1] Muwonge H, Kikomeko S, Sembajjwe LF (2013). How Reliable Are Hematological Parameters in Predicting Uncomplicated Plasmodium falciparum Malaria in an Endemic Region?. ISRN Trop Med.

[2] Philipose CS, Umashankar T (2016). The role of haematological parameters in predicting malaria with special emphasis on neutrophil lymphocyte count ratio and monocyte lymphocyte ratio: A single Institutional experience. Trop Parasitol.

[3] Liu W, Li Y, Shaw KS (2014). African origin of the malaria parasite Plasmodium vivax. Nat Commun.

[4] Alam MT, Bora H, Bharti PK (2007). Similar trends of pyrimethamine resistance-associated mutations in Plasmodium vivax and P. falciparum. Antimicrob Agents Chemother.

[5] Pukrittayakamee S, Imwong M, Looareesuwan S (2004). Therapeutic responses to antimalarial and antibacterial drugs in vivax malaria. Acta Trop.

[6] Baird JK (2004). Chloroquine resistance in Plasmodium vivax. Antimicrob Agents Chemother.

[7] Imwong M, Pukrittayakamee S, Renia L (2003). Novel point mutations in the dihydrofolate reductase gene of Plasmodium vivax: evidence for sequential selection by drug pressure. Antimicrob Agents Chemother.

[8] Malkin EM, Diemert DJ, McArthur JH (2005). Phase 1 clinical trial of apical membrane antigen 1: an asexual blood-stage vaccine for Plasmodium falciparum malaria. Infect Immun.

[9] Saul A, Lawrence G, Allworth A (2005). A human phase 1 vaccine clinical trial of the Plasmodium falciparum malaria vaccine candidate apical membrane antigen 1 in Montanide ISA720 adjuvant. Vaccine.

[10] Morais CG, Soares IS, Carvalho LH (2006). Antibodies to Plasmodium vivax apical membrane antigen 1: persistence and correlation with malaria transmission intensity. Am J Trop Med Hyg.

[11] Remarque EJ, Faber BW, Kocken CH (2008). Apical membrane antigen 1: a malaria vaccine candidate in review. Trends Parasitol.

[12] Bannister LH, Hopkins JM, Dluzewski AR (2003). Plasmodium falciparum apical membrane antigen 1 (PfAMA-1) is translocated within micronemes along subpellicular microtubules during merozoite development. J Cell Sci.

[13] Healer J, Murphy V, Hodder AN (2004). Allelic polymorphisms in apical membrane antigen-1 are responsible for evasion of antibody-mediated inhibition in Plasmodium falciparum. Mol Microbiol.

[14] Narum DL, Thomas AW (1994). Differential localization of full-length and processed forms of PF83/AMA-1 an apical membrane antigen of Plasmodium falciparum merozoites. Mol Biochem Parasitol.

[15] Howell SA, Well I, Fleck SL (2003). A single malaria merozoite serine protease mediates shedding of multiple surface proteins by juxtamembrane cleavage. J Biol Chem.

[16] Pizarro JC, Vulliez-Le Normand B, Chesne-Seck ML (2005). Crystal structure of the malaria vaccine candidate apical membrane antigen 1. Science.

[17] Kocken CH, Withers-Martinez C, Dubbeld MA (2002). High-level expression of the malaria blood-stage vaccine candidate Plasmodium falciparum apical membrane antigen 1 and induction of antibodies that inhibit erythrocyte invasion. Infect Immun.

[18] Triglia T, Healer J, Caruana SR (2000). Apical membrane antigen 1 plays a central role in erythrocyte invasion by Plasmodium species. Mol Microbiol.

[19] Polley SD, Conway DJ (2001). Strong diversifying selection on domains of the Plasmodium falciparum apical membrane antigen 1 gene. Genetics.

[20] Escalante AA, Grebert HM, Chaiyaroj SC (2001). Polymorphism in the gene encoding the apical membrane antigen-1 (AMA-1) of Plasmodium falciparum. X. Asembo Bay Cohort Project. Mol Biochem Parasitol.

[21] Cortes A, Mellombo M, Mueller I (2003). Geographical structure of diversity and differences between symptomatic and asymptomatic infections for Plasmodium falciparum vaccine candidate AMA1. Infect Immun.

[22] Crewther PE, Matthew ML, Flegg RH (1996). Protective immune responses to apical membrane antigen 1 of Plasmodium chabaudi involve recognition of strain-specific epitopes. Infect Immun.

[23] Renia L, Ling IT, Marussig M (1997). Immunization with a recombinant C-terminal fragment of Plasmodium yoelii merozoite surface protein 1 protects mice against homologous but not heterologous P. yoelii sporozoite challenge. Infect Immun.

[24] Sadrizadeh B (2001). Malaria in the world, in the eastern Mediterranean region and in Iran: review article WHO/EMRO. Report.

[25] Esmaeili Rastaghi AR, Nedaei F, Nahrevanian H (2014). Genetic diversity and effect of natural selection at apical membrane antigen-1 (AMA-1) among Iranian Plasmodium vivax isolates. Folia Parasitol.

[26] Tamura K, Dudley J, Nei M (2007). MEGA4: Molecular Evolutionary Genetics Analysis (MEGA) software version 40. Mol Biol Evol.

[27] Librado P, Rozas J (2009). DnaSP v5: a software for comprehensive analysis of DNA polymorphism data. Bioinformatics.

[28] Thakur A, Alam MT, Bora H (2008). Plasmodium vivax: sequence polymorphism and effect of natural selection at apical membrane antigen 1 (PvAMA1) among Indian population. Gene.

[29] Rajesh V, Elamaran M, Vidya S (2007). Plasmodium vivax: genetic diversity of the apical membrane antigen-1 (AMA-1) in isolates from India. Exp Parasitol.

[30] Putaporntip C, Jongwutiwes S, Grynberg P (2009). Nucleotide sequence polymorphism at the apical membrane antigen-1 locus reveals population history of Plasmodium vivax in Thailand. Infect Genet Evol.

[31] Ord RL, Tami A, Sutherland CJ (2008). ama1 genes of sympatric Plasmodium vivax and P falciparum from Venezuela differ significantly in genetic diversity and recombination frequency. PLoS One.

[32] Gunasekera AM, Wickramarachchi T, Neafsey DE (2007). Genetic diversity and selection at the Plasmodium vivax apical membrane antigen-1 (PvAMA-1) locus in a Sri Lankan population. Mol Biol Evol.

[33] Snow RW, Marsh K (1998). New insights into the epidemiology of malaria relevant for disease control. Br Med Bull.

[34] Kleinschmidt I, Sharp B (2001). Patterns in age-specific malaria incidence in a population exposed to low levels of malaria transmission intensity. Trop Med Int Health.

[35] Danis-Lozano R, Rodriguez MH, Betanzos-Reyes AF (2007). Individual risk factors for Plasmodium vivax infection in the residual malaria transmission focus of Oaxaca, Mexico. Salud Publica Mex.

[36] Baird JK, Masbar S, Basri H (1998). Age-dependent susceptibility to severe disease with primary exposure to Plasmodium falciparum. J Infect Dis.

[37] Gupta S, Snow RW, Donnelly CA (1999). Immunity to non-cerebral severe malaria is acquired after one or two infections. Nat Med.

[38] Kuntz ID (1972). Protein folding. J Am Chem Soc.

[39] Rose GD (1978). Prediction of chain turns in globular proteins on a hydrophobic basis. Nature.

[40] Hodder AN, Crewther PE, Matthew ML (1996). The disulfide bond structure of Plasmodium apical membrane antigen-1. J Biol Chem.

